# High resolution structure of an M23 peptidase with a substrate analogue

**DOI:** 10.1038/srep14833

**Published:** 2015-10-06

**Authors:** Maja Grabowska, Elzbieta Jagielska, Honorata Czapinska, Matthias Bochtler, Izabela Sabala

**Affiliations:** 1 International Institute of Molecular and Cell Biology, Ks. Trojdena 4, 02-109 Warsaw, Poland; 2 Institute of Biochemistry and Biophysics, Pawinskiego 5a, 02-106 Warsaw, Poland

## Abstract

LytM is a *Staphylococcus aureus* autolysin and a homologue of the *S. simulans* lysostaphin. Both enzymes are members of M23 metallopeptidase family (MEROPS) comprising primarily bacterial peptidoglycan hydrolases. LytM occurs naturally in a latent form, but can be activated by cleavage of an inhibitory N-terminal proregion. Here, we present a 1.45 Å crystal structure of LytM catalytic domain with a transition state analogue, tetraglycine phosphinate, bound in the active site. In the electron density, the active site of the peptidase, the phosphinate and the “diglycine” fragment on the P1′ side of the transition state analogue are very well defined. The density is much poorer or even absent for the P1 side of the ligand. The structure is consistent with the involvement of His260 and/or His291 in the activation of the water nucleophile and suggests a possible catalytic role for Tyr204, which we confirmed by mutagenesis. Possible mechanisms of catalysis and the structural basis of substrate specificity are discussed based on the structure analysis.

LytM belongs to M23 family of zinc dependent metallopeptidases (MEROPS^1^), which comprises M23A family proteins, represented by *P. aeruginosa* LasA, and M23B family proteins, including LytM and lysostaphin. LytM was originally identified in an autolysis defective mutant of *S. aureus*^2^, and like the other M23B family proteins, has glycylglycine endopeptidase activity. LytM cleaves staphylococcal peptidoglycan *in vitro* and acts as an autolysin *in vivo*^2^. Other roles for the protein have also been proposed, e.g. vancomycine resistance^3^ or the release of protein A from the cell walls of *S. aureus*^4^.

Over the past years, many members of the M23 metallopeptidase family have been identified and biochemically characterized. Structures have been determined for some of them, e.g. LytM^5^,^6^, LasA^7^ and recently lysostaphin, a prototypic enzyme of the M23B group and the best studied bacteriocin^8^. LytM is a two-domain protein: the N-terminal domain is preceded by a signal peptide, the C-terminal domain can be divided into an occluding region and a region of high similarity to the lysostaphin catalytic domain (52% amino acid identity over 106 residues). In the full length LytM structure, a Zn^2+^ cation is tetrahedrally coordinated by the side chains of N117, H210, D214 and H293. The N117 residue of the occluding region blocks part of the active site cleft and occupies a coordination site for the substrate carbonyl oxygen. Therefore, proteolytic processing of LytM is required for its activation at least *in vitro*. This latency mechanism has been termed “asparagine switch”^5^ by analogy to the cysteine switch regulating matrix metallopeptidases^9^.

Several crystal forms of either full length LytM and its analogues or their catalytic domains have been described before. However the substrate/inhibitor complex of an enzyme of this class remained elusive. Therefore details of the catalytic mechanism and the structural basis of substrate specificity have remained unclear. We have addressed these issues by a structural analysis of LytM catalytic domain in complex with transition state analogue. The findings may also be helpful for attempts to modify the substrate specificity of these biotechnologically and medically relevant enzymes. Many of the M23 enzymes, in particular lysostaphin, display potent bacteriolytic activity also against antibiotic resistant strains, which find use in experimental treatment of infected patients, elimination of *S. aureus* in asymptomatic carriers, eradication of biofilms from artificial surfaces or antibacterial catheter coatings, and other applications^10^,^11^,^12^,^13^,^14^.

## Results and Discussion

### Co-crystal structure

**Figure 1:**
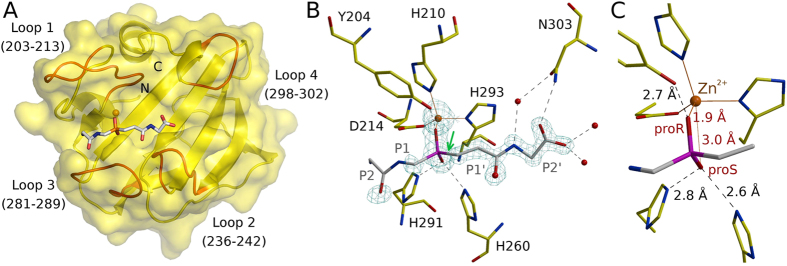
The binding mode of the phosphinate. (**A**) The overall structure of the LytM catalytic domain in complex with the transition state analogue. The loops lining the LytM substrate binding cleft are indicated in red. (**B**) The interactions of the transition state analogue with the protein. The map is the composite omit map contoured at 2.0 Å rmsd. (**C**) Detail of the phosphinate binding mode (only the P1 and P1′residues excluding the carbonyl are shown). Note that the proR and proS assignment is according to IUPAC priorities for the transition state and not for the phosphinate itself.

Crystals of LytM in complex with tetraglycine phosphinate were monoclinic (space group *P*2_1_) and contained four LytM-phosphinate complexes in the asymmetric unit. They diffracted to 1.45 Å on a synchrotron beamline with average B-factor 16.7 Å^2^ (according to the XDS calculated Wilson plot^15^). The structure was solved by molecular replacement using the structure of LytM (2B0P) and refined to final R_work_ and R_free_ factors of 14.9% and 18.5%, respectively (Fig. 1A and Suppl. Table 1). All four LytM copies in the asymmetric unit were very similar (all atom rmsds of around 0.6 Å), even though NCS restraints were not used for refinement. The ligand density was very strong for the phosphinate phosphorus and oxygen atoms, and also for the “diglycine” on the primed side (downstream of the scissile bond/phosphinate, Schechter and Berger nomenclature^16^). In contrast, the density was weak or absent on the non-primed site (upstream of the scissile bond/phosphinate) and this part of the ligand was modeled only tentatively (Fig. 1B). Average B was lowest for the protein (13 Å^2^), slightly higher for the primed site and P1 residue of the phosphinate (17 Å^2^) and highest (40 Å^2^) for the P2 residue of the phosphinate (B-factors were calculated for full occupancy and single conformation of the phosphinate and then the alternatives were averaged).

### Local phosphinate environment, implications for catalysis

**Figure 2:**
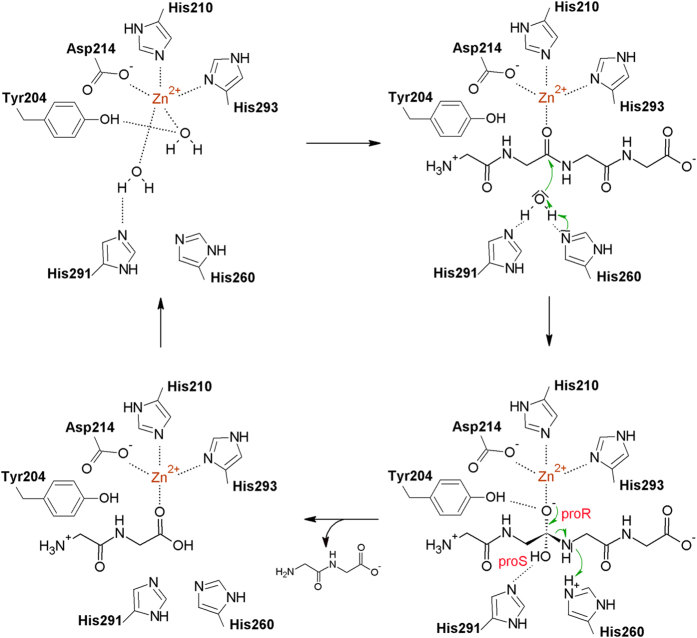
Proposed catalytic mechanism of the LytM class of enzymes. The top left and right bottom panels are based on the actual crystal structures of LasA (3IT5) and LytM (presented here) except that in the complex with the phosphinate His260 is not protonated, and binds the proS oxygen atom instead. The other two panels are models and therefore only the most convincing interactions have been shown but it is possible that Tyr204, His260 and His291 would contribute to the substrate/product binding as well. The contact between the water coordinating Zn^2+^ ion and the Tyr204 is observed in some structures (3IT5) but not in others (4QPB), and must be weak so that the substrate can displace the water molecule. The LytM numbering has been used in all panels.

The tetraglycine phosphinate mimics the transition state of pentaglycine crossbridge hydrolysis. We use proR and proS labels for the oxygen atoms according to the IUPAC rules applied to the transition state intermediate (and not the phosphinate itself) to make the terminology independent of the analogue used to elucidate mechanism. In this convention, the proR and proS oxygen atoms are 1.9 and 3.0 Å away from the Zn^2+^ cation, respectively (Fig. 1C). The observed distances suggest identification of the transition state proR oxygen atom with the carbonyl oxygen atom of the substrate and of the proS oxygen atom with the incoming water molecule or hydroxide ion. This implies polarization of the scissile amide bond due to the coordination of the carbonyl oxygen atom by the Zn^2+^ ion in the active site, as in almost all detailed proposals for catalysis by single cation metallopeptidases^17^,^18^. It also implies that the nucleophilic water molecule or hydroxide ion has to approach from the *si* face of the amide bond (Fig. 2). In this scenario, the metal ion is not involved in the activation of the nucleophile, as proposed for some but not all metallopeptidases^17^,^18^. Instead, the structure suggests that the incoming water molecule/hydroxide ion is activated by His260 and His291, as tentatively already proposed in the case of LytM^19^, and for the equivalent histidines also in the case of the LytM orthologue LasA^7^. In the experimental structure of the phosphinate complex, the oxygen atom that would correspond to the proS oxygen of the transition state is 2.6 and 2.8 Å away from the Nε atoms of His260 and His291, respectively (Fig. 1C). Loss of activity when either histidine is replaced by alanine in LytM^5^,^19^ (or its lysostaphin^20^, LasA^21^ and Ale-1^22^ homologues) supports a role of the histidines in catalysis.

**Figure 3:**
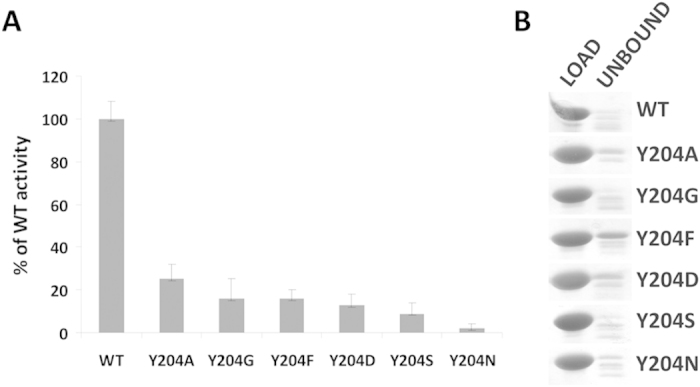
Mutant analysis. (**A**) Activity assay. The activities of Tyr204 mutants were tested in lytic assay and compared to the activity of the wild type enzyme. *S. aureus* cells collected at exponential phase were suspended in lysis buffer (50 mM glycine, pH 8.0) to an apparent OD_600_ ~ 1.0, treated with LytM and its mutants (at 100 nM final concentration) and incubated at room temperature for 60 min. The final readout of OD was done in triplicates and the experiment was repeated three times. (**B**) Binding assay. Samples of purified proteins (20 μg) mixed with purified *S. aureus* peptidoglycans suspended in 50 mM glycine buffer (pH 8.0) were incubated for 15 min on ice. Insoluble peptidoglycans with bound proteins and supernatant were separated by centrifugation. Control proteins (load) and supernatant with unbound proteins were separated by SDS-PAGE and stained with Coomassie. Fractions of proteins bound to peptidoglycans generated a smear on SDS-PAGE and are not shown in the figure.

**Figure 4:**
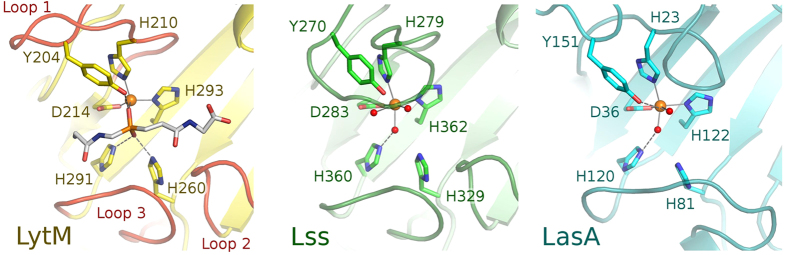
Active site of LytM and related proteins: LytM (current structure), Lss—lysostaphin from *Staphylococcus simulans* (4QPB), LasA from *Pseudomonas aeruginosa* (3IT5). Orange spheres—zinc ions, red spheres—water molecules coordinating zinc ions.

The co-crystal structure of LytM with the phosphinate does not only clarify the role of residues in the active site previously suspected to be involved in catalysis. It also suggests that a residue not previously implicated in LytM catalysis, Tyr204, may make an important contribution as it was suggested for a corresponding residue in LasA^7^. The hydroxyl group of Tyr204 donates a hydrogen bond to the oxygen atom of the phosphinate that corresponds to the proR oxygen atom of the transition state, which we have argued is derived from the scissile amide bond. In the absence of an experimental structure of LytM with a substrate, we cannot be sure where the carbonyl would be located in the Michaelis complex, but the interpretation of proS and proR oxygens suggests that it would be located between these atoms. If this deduction is correct, then the tyrosine is better positioned to interact with the substrate carbonyl oxygen atom in the transition state than in the ground state. In other words, Tyr204 would be expected to make a significant contribution to catalysis, somewhat akin to the hydrogen bond donors contributing to the oxyanion binding in serine peptidases, which also stabilize the tetrahedral intermediate of the reaction^23^. Mutational analysis confirmed the significance of Tyr204 for LytM catalysis. We have generated a set of Tyr204 mutants (glycine, alanine, serine, phenylalanine, aspartate and asparagine) and tested them in binding and lytic activity assays. In all tested mutants the activity of the enzyme was very weak or even completely abolished (Fig. 3A) while peptidoglycan binding remained almost unchanged (Fig. 3B). Interestingly, a tyrosine is found in spatially equivalent position also in LasA, even though it is located elsewhere in the amino acid sequence (Fig. 4). LytM and LasA are representative in this respect. In LytM and other closely related enzymes of the M23B subfamily, the tyrosine is always present close to the N-terminal end, within loop 1 (for a nomenclature of loops, see Firczuk *et al.*, 2005^6^ and Spencer *et al.*, 2010^7^). In LasA and other M23A family enzymes, the structurally equivalent tyrosine is located in the C-terminal part of the sequence. Convergent evolution of a tyrosine in proximity to the proR oxygen atom of the transition state further supports a functional role of this tyrosine residue.

### Specific protein-transition state analogue interactions

The tetraglycine phosphinate makes surprisingly few specific contacts with the LytM protein. Only its well-ordered, prime side participates in the interactions (Fig. 1B). The NH group of the P2′ glycine is involved in a water mediated hydrogen bond to the carboxamide oxygen atom of Asn303. A hydrogen bond with good geometry is also possible from the carboxamide nitrogen of Asn303 to the terminal carboxylate group of the ligand. In a natural peptidoglycan substrate, the peptide chain would be extended in this position, depending on the register of cleavage in the pentaglycine crossbridge either by another glycine, or by an amide bond to the stem peptide. In such an arrangement, the hydrogen bond from the Asn303 side chain to the carbonyl oxygen atom could still be formed. The LytM crystal structure also shows that there is space to continue the polypeptide chain beyond the carboxylate of the transition state analogue.

The P1′ glycine of the phosphinate is not involved in any direct hydrogen bonding contacts with the protein. Its carbonyl oxygen atom is in water mediated contact with side chain of Asn286 and main chain of Tyr239. In the experimentally studied phosphinate analogue of the transition state, a methylene group replaces the NH group of the transition state. We cannot exclude that this exchange, which alters hydrogen bonding properties and geometry might let us miss interactions that could occur in the actual transition state.

In general, the scarcity of favorable interactions between the tetraglycine phosphinate and LytM is not unexpected. The lack of side chains in the substrate limits the opportunities for specific interactions. The limited number of backbone contacts lets one wonder whether “backwards” bound substrates might also be cleaved. We cannot formally exclude this possibility, but consider it unlikely because the phosphinate transition state analogue binds in the same orientation in all four independent instances in the asymmetric unit of the crystal. Moreover, “backwards” bound peptides, typically derived from proregions of zymogens, tend to act as peptidase inhibitors and not substrates, at least in the case of cysteine peptidases^24^,^25^.

### Structural constraints on the amino acid sequence

As the phosphinate is likely to approximate the transition state of the reaction, it can be used to derive structural constraints for the amino acid sequence of substrate peptides. On the non-primed side of the phosphinate, the electron density is too ambiguous for such analysis (Fig. 1B). On the primed side, the computational replacement of P1′ propionyl residue is possible (Suppl. Fig. 1). Tyr204 and His210 are both sufficiently far away to avoid clashes. Near the computationally introduced Cβ atom on this residue, there are two clearly defined water molecules in the experimental structure (at distances of 2.5 Å and 3.1 Å, respectively). Thus, there is substantial space for bulkier side chains, which could displace the water molecules (an entropically favored process). We conclude that a stringent selection of the P1′ residue is not expected based on the structure, at least not by steric exclusion. Conclusions are different for the next residue downstream. When a Cβ-amino acid is computationally grafted onto this residue, it comes too close (2.7 Å) to the main chain of Gly241. A comparison with previous LytM structures shows moderate flexibility of the loop containing Gly241, which is not sufficient to relieve the clash. Hence, we expect that steric exclusion contributes to selectivity for the P2′ glycine residue (Suppl. Fig. 1).

### Comparison with previously observed binding modes of glycine rich loop

**Figure 5:**
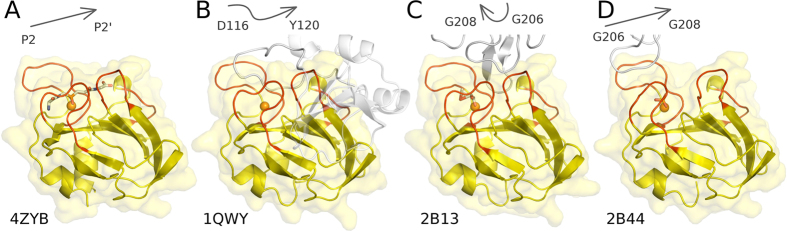
Peptides and analogues in the substrate binding groove of LytM. (**A**) LytM catalytic domain with the phosphinate transition state analogue; (**B**) full length LytM with active site blocked by peptide from occluding region (PDB 1QWY); (**C**,**D**) catalytic domain of LytM with glycine rich loop from neighboring molecule wedged into the active site cleft (*P*4_1_ (2B13) and *P*3_2_21 (2B44) crystal forms). Arrows mark the direction of the main chain in the transition state analogue and protein fragments occupying in the cleft.

LytM happens to contain a surface exposed loop distant from the active site with 3 consecutive glycine residues (Gly206-Gly208). In two previous crystal forms, this loop comes close to the active site of another LytM molecule, so that it might mimic the binding of a substrate^6^. However, it was already noticed in the previous work that the glycine-rich loop was running through the active site region in opposite directions in different crystal forms (Fig. 5). Moreover, the resolution was sufficient to conclude that the loop was not cleaved in the “incidental” crystallographic contacts, and therefore could not mimic the binding mode of a substrate accurately^6^. We have now revisited the previously observed binding modes in the light of the experimental density for the phosphinate. The glycine rich loop of a neighboring molecule in the *P*4_1_ crystal form (PDB: 2B13) runs indeed through the region of the active site cleft also occupied by the well-defined part of the phosphinate, but the direction of the polypeptide chain is reversed (Fig. 5C). The loop from the neighboring molecule in the *P*3_2_21 crystal form (PDB: 2B44) runs in the correct direction, but is very significantly displaced relative to the phosphinate (Fig. 5D). We conclude from this analysis that the crystallographic packing effects that were noticed earlier did correctly identify the active site cleft, but did not accurately mimic the substrate binding mode.

### Comparison with binding mode of the inhibitory N-terminal proregion

**Figure 6:**
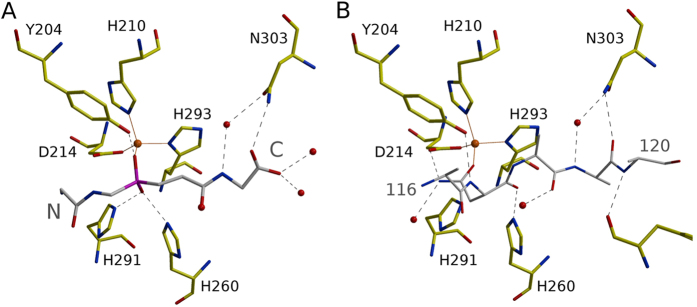
Transition state analogue and inhibitory proregion in the LytM substrate binding groove. (**A**) Tetraglycine phosphinate and (**B**) proregion (PDB 1QWY).

LytM is naturally made as a latent enzyme with an inhibitory region at its N-terminus, which can be cleaved off even by physiologically unrelated peptidases^5^. Although such LytM activation in physiological circumstances has never been shown, this region nevertheless behaves biochemically much like a proregion and will be called so in the following. In the previously determined structure of full length LytM^5^ the proregion runs through the active site cleft, but is not cleaved (Fig. 5B). This can be attributed to a relatively large distance of the peptide backbone from the active site Zn^2+^ ion. Additionally the Zn^2+^ ion is trapped in a tetrahedral coordination arrangement with the asparagine carboxamide oxygen atom as the fourth ligand in addition to the three usual ones (His210, Asp214, His293). The current structure makes it possible to relate the proregion and substrate (analogue) binding modes. We found a surprising similarity between the P1′ side of the phosphinate and the 117-NND—119 fragment of the proregion (Fig. 6). Both run in the same direction with the distance between corresponding Cα-atoms below 2.0 Å. The agreement does not hold for the phosphinate group (corresponding to the scissile bond). Whereas the phosphinate runs straight through the active site, the proregion kinks sharply. This kink provides sufficient distance for the “asparagine switch” Asn117 residue to contact the metal via the carbonyl oxygen atom of the side and not main chain. We conclude from this analysis that the proregion of LytM mimics a substrate in the primed, but not in the non-primed region of the substrate binding cleft.

### Comparison of the substrate binding modes in the LytM/lysostaphin and LasA families

LytM belongs to the same group of evolutionarily related peptidases as lysostaphin and LasA (MEROPS family M23). The binding mode of peptides to LasA has been investigated in detail by computational studies. The substrate binding cleft of the enzyme is lined by four loops, which are conserved also in lysostaphin and LytM. The catalytic Zn^2+^ ion is located at the bottom of this cleft. Based on computational results Spencer and colleagues^7^ have concluded that LasA binds its substrates in an orientation that puts the N-terminus closer to loops 1 and 3 and the C-terminus closer to loops 2 and 4.

The location of the binding cleft and substrate orientation predicted for LasA agree with the phosphinate binding mode presented here, supporting the generality of the broader conclusions from this work. However, the details of substrate binding are distinctly different: while the tetraglycine phosphinate in LytM adopts an extended, β-strand like structure, the trace of the modeled LasA substrate peptide chain is more irregular, and in some cases, amide bonds in adjacent residues are oriented perpendicular to each other^7^. When the LasA substrate is modelled according to the binding mode of the phosphinate to LytM, the LasA specificity (in particular the preference for a P1′ phenylalanine residue) remains unexplained. Therefore, we conclude that the detailed differences in the binding modes of LytM and LasA are likely genuine and not modeling/experiment artifacts.

## Conclusions

The structure of LytM with a tetraglycine phosphinate transition state analogue reveals details of the catalytic mechanism. It supports the role of His260 and His291 in activation of the nucleophilic water molecule or hydroxide ion, and suggests a previously unrecognized catalytic role for Tyr204. The tyrosine may help by hydrogen bonding to stabilize the oxyanion intermediate of the reaction (akin to the role of the oxyanion hole in serine peptidases). A catalytic function of Tyr204, which is structurally conserved in the entire M23 family, is also supported by site-directed mutagenesis experiments. The LytM phosphinate co-crystal structure also helps to identify the structural determinants of substrate recognition and binding.

## Methods

### Protein purification

The catalytic domain of LytM (residues 185–316) was cloned into expression plasmid (pET15b) under the control of the T7 promoter and lac repressor as described previously^6^. For protein expression, transformed BL21(DE3) *E. coli* cells were grown in autoinductive medium (Auto Induction Medium LB Broth Base including trace elements, Formedium, UK) for 24 hours at 30 °C. Cells were harvested and resuspended in buffer A (20 mM Tris-HCl, pH 7.5, 50 mM NaCl). After cell disruption by Constant Cell Disruption System (Constant System Ltd., UK) and clarification of the lysate, the supernatant was applied to a SP Sepharose FF column (GE Healthcare, Sweden) equilibrated in buffer A. The protein recovered in the flow through was 90% pure. After concentration, the protein was subjected to gel filtration step on Superdex 75 column (GE Healthcare, Sweden) in buffer A.

### Crystallization and structure determination

Several different crystal forms of LytM catalytic domain have been reported before^6^. In this work, we found an additional crystallization condition, which led to strongly diffracting crystals in space group *P*2_1_. Catalytic domain of LytM (12 mg ml^−1^) was mixed in 1:1 ratio with the reservoir buffer containing 50 mM HEPES-CsOH pH 7.5, 200 mM calcium chloride and 25% polyethylene glycol 4000. Crystallization experiments were carried out by the vapor diffusion method in sitting drops at 17 °C. Crystals grew during a month of equilibration against the reservoir buffer.

The datasets were measured at the 14.1 and 14.2 beamlines of BESSY synchrotron (Berlin, Germany). The best crystals diffracted to about 1.2 Å. Data was processed with the help of XDS software^26^. The structure was readily interpreted by molecular replacement using previously published LytM catalytic domain structure^6^ with the help of MolRep^27^ and ARP/wARP^28^ programs. The crystals contained four molecules in the asymmetric unit. In each of the molecules the active site was solvent accessible and allowed for the ligand binding.

Unfortunately, attempts to co-crystallize LytM catalytic domain with substrate analogues, including tetraglycine, glycine hydroxamate and short peptides, such as GGSGG or GGA, all led to electron densities in which the ligand was either altogether absent or very poorly defined. Findings were very similar when the analogues were not added to the crystallization mix, but instead soaked into crystals. We reasoned that the failure to obtain complexes might be related to the very poor activity of LytM in “high salt” buffers. Therefore, we soaked crystals of the newly identified crystal form in low salt (low conductivity) conditions where 50–60 mM substrate analogue solution in 30% polyethylene glycol 4000 (0.5 μl) was added to crystallization drop (0.2 μl). Crystals were then harvested and cryoprotected in the same conditions at various time points ranging from minutes to 4 weeks of incubation. Most of the tested crystals had an additional density in the active center, but only the incubation with phosphinic derivative of tetraglycine (H-Gly-Glyψ[P(O)(OH)CH_2_]-Gly-Gly-OH) led to the diffraction data quality that allowed unquestionable identification of the ligand. The structure was refined with REFMAC^29^ and PHENIX^30^ and manually improved in COOT^31^. Data and refinement statistics are summarized in Suppl. Table 1. The atomic coordinates of the final model and the corresponding structure factors have been deposited in the Protein Data Bank (accession code 4ZYB).

### Mutagenesis

LytM catalytic domain mutants: Y204A, Y204D, Y204F, Y204G, Y204N and Y204S were generated by PCR-based site-directed mutagenesis according to the manufacturer protocol with Phusion high-fidelity DNA polymerase (Thermo Scientific, USA) using pET15b-LytMCD plasmid as a PCR template. In order to confirm that only the intended mutations were introduced, all of the DNA constructs of the mutants were sequenced. The wild-type protein and mutants were overexpressed with an N-terminal histidine tag as described above. The collected cells were harvested and the lysate was used for protein purification. The first purification step was performed on a 5 ml precharged Ni Sepharose^TM^ High Performance HisTrap^TM^ HP column (GE Healthcare, Sweden). The imidazole gradient was run from 50 mM to 1 M in buffer B (20 mM Tris-HCl pH 7.0, 200 mM NaCl). Histidine-tagged LytM mutants eluted at 300–400 mM imidazole. After concentration, the proteins were subjected to gel filtration step on Superdex 75 column (GE Healthcare, Sweden) in buffer B supplemented with 10% glycerol as a final purification step.

### Cell lysis assay

*S. aureus* 8325–4 strain cells collected at the exponential growth phase were washed and suspended in buffer C (50 mM glycine, pH 8.0) to an apparent OD_595_ of 0.8–1.0. Enzymes were added to the final concentration of 100 nM and 200 μl of reaction transferred onto the microtiter plate. Plates were incubated at room temperature and OD of the suspension was checked at the wavelength of 595 nm every 10 min for 1 hour with 5 sec shaking. Lytic activity was calculated as a percentage of initial OD_595_. Each experiment was repeated three times in triplicates.

### Peptidoglycan binding assay

The binding of proteins to peptidoglycans (PG) purified according to previously described protocol^5^ was observed following incubation of 10 μg protein with peptidoglycan at OD_600_ = 20 in 50 μl of buffer C at 4 °C for 15 minutes. Mixtures were separated by centrifugation into bound (pellet) and unbound (supernatant) fractions which were then analyzed on 15% SDS-PAGE gels.

## Additional Information

**How to cite this article**: Grabowska, M. *et al.* High resolution structure of an M23 peptidase with a substrate analogue. *Sci. Rep.* **5**, 14833; doi: 10.1038/srep14833 (2015).

## Supplementary Material

Supplementary Information
