# Supplementary material for: High resolution structure of an M23 peptidase with a substrate analogue
Source: Sci Rep. 2015 Oct 6;5:14833. doi: 10.1038/srep14833 (PMC4594094; doi:10.1038/srep14833)

Supplementary material for:

High resolution structure of an M23 peptidase with a substrate analogue

Maja Grabowska[1], Elzbieta Jagielska[1], Honorata Czapinska[1,2], Matthias Bochtler[1,2*], and Izabela Sabala[1*]

[1]International Institute of Molecular and Cell Biology, Ks. Trojdena 4, 02-109 Warsaw, Poland

[2]Institute of Biochemistry and Biophysics, Pawinskiego 5a, 02-109 Warsaw, Poland

[*]Corresponding coauthors:

Izabela Sabala e-mail: izabela@iimcb.gov.pl

Matthias Bochtler e-mail: mbochtler@iimcb.gov.pl

Supplementary Table 1. Data collection and refinement statistics.

Data collection statistics	
Space group	$P2_1$
Cell dimensions	
a (Å)	32.0
b (Å)	101.6
c (Å)	77.8
β (°)	91.5
Beamline	BESSY 14.1
Wavelength (Å)	0.917
Resolution (Å)<br>lowest shell<br>highest shell	40-1.45<br>40-4.32<br>1.54-1.45
R_{sym} (%)*	11.2 (3.9, 64.9)
R_{meas} (%)*	12.1 (4.2, 70.6)
$CC_{1/2}$*	99.8 (99.9, 81.3)
$I/\sigma I$*	12.0 (39.5, 2.5)
Completeness (%)*	99.7 (99.9, 98.1)
Multiplicity*	6.8 (6.9, 6.5)
V_M	2.2
Solvent content (%)	44
Refinement statistics	
No. reflections	87615
R_{work}/ R_{free}	14.9 / 18.5
No. atoms** (average B [$Å^2$])	5713 (15.5)
Protein	4678 (13.1)
Tetraglycine phosphinate	87 (22.1***)
Other	948 (27.1)
R.m.s deviations	
Bond lengths (Å)	0.006
Bond angles (°)	1.1
Ramachandran<br>allowed region (%)<br>favored region (%)	 100.0<br>97.8
Molprobity clashscore	1.2

* Lowest and highest shell in brackets

** Alternative conformations counted separately.

*** Tetraglycine phosphinate B-factors correspond to the average values for alternative conformations refined separately with full occupancy.

Supplementary Figure 1. **Binding mode of tetraglycine phosphinate to the active site cleft of LytM. (A)** Overall structure of the superposed four LytM-phosphinate molecules present in the asymmetric unit. The protein, transition state analogue and the zinc ion are colored according to their B-factors, from blue (<5 Å^2) to red (>50 Å^2). **(B)** LytM active site showing all observed conformations of the analogue and binding cleft lining loops. **(C)** The positions of the CB atoms have been modelled for the well defined P1' and P2' residues of the tetraglycine phosphinate. Potential contacts/steric clashes with the protein residues and waters that would be displaced by alanines instead of glycines are marked by dotted lines (assuming a 3.5 Å distance cutoff).

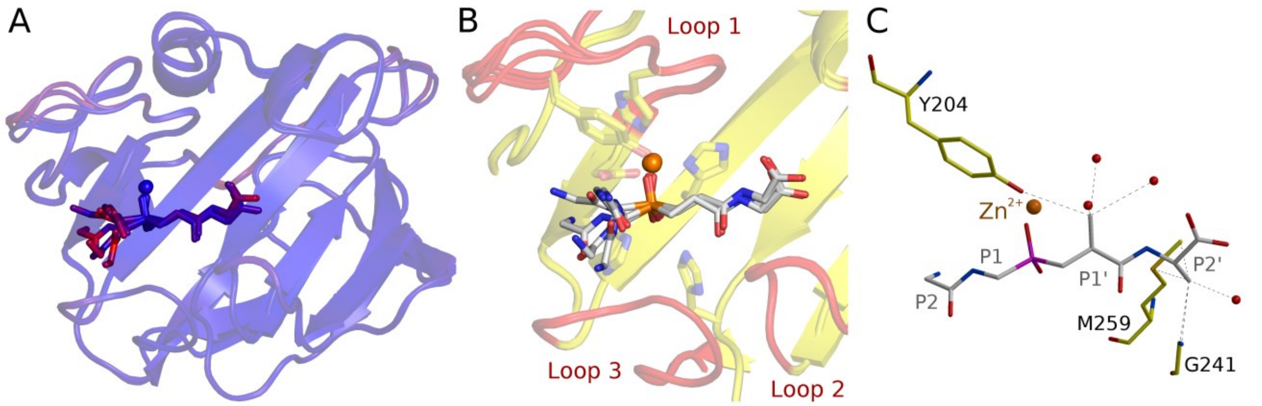

Supplement: Supplementary Information [file srep14833-s1.pdf]
